# Hill-Climbing search and diversification within an evolutionary approach to protein structure prediction

**DOI:** 10.1186/1756-0381-4-23

**Published:** 2011-07-30

**Authors:** Camelia Chira, Dragos Horvath, D Dumitrescu

**Affiliations:** 1 Computer Science Department, Babes-Bolyai University, 1 Kogalniceanu, Cluj-Napoca 400084, Romania; 2 Laboratoire d'Infochimie, UMR 7177, University Strasbourg, France

## Abstract

Proteins are complex structures made of amino acids having a fundamental role in the correct functioning of living cells. The structure of a protein is the result of the protein folding process. However, the general principles that govern the folding of natural proteins into a native structure are unknown. The problem of predicting a protein structure with minimum-energy starting from the unfolded amino acid sequence is a highly complex and important task in molecular and computational biology. Protein structure prediction has important applications in fields such as drug design and disease prediction. The protein structure prediction problem is NP-hard even in simplified lattice protein models. An evolutionary model based on hill-climbing genetic operators is proposed for protein structure prediction in the hydrophobic - polar (HP) model. Problem-specific search operators are implemented and applied using a steepest-ascent hill-climbing approach. Furthermore, the proposed model enforces an explicit diversification stage during the evolution in order to avoid local optimum. The main features of the resulting evolutionary algorithm - hill-climbing mechanism and diversification strategy - are evaluated in a set of numerical experiments for the protein structure prediction problem to assess their impact to the efficiency of the search process. Furthermore, the emerging consolidated model is compared to relevant algorithms from the literature for a set of difficult bidimensional instances from lattice protein models. The results obtained by the proposed algorithm are promising and competitive with those of related methods.

## Introduction

Proteins are complex, irregular structures playing key roles in many cellular functions [1,2]. A protein molecule is a chain of amino acids linked together by peptide bonds (the primary structure), which tend to locally adopt some few characteristic conformations or form flexible loops (the secondary level structure). The function of a protein mainly depends on the tertiary structure which represents the relative arrangement of its secondary structure elements. An open protein chain normally folds into a three-dimensional configuration (called native state) to perform its function. The knowledge generated by a correct prediction of protein tertiary structures is of huge importance for many applications fields including drug design and disease prediction [3].

Protein structure prediction refers to the problem of predicting the tertiary structure of a protein based on its primary structure information. This is a computationally challenging problem of significant importance in biochemistry, molecular biology and biophysics. Starting from an unfolded chain of amino acids, protein folding simulations aim to find a final protein structure having minimum energy. Detecting such a structure represents an NP-hard problem [1,4] even in simplified lattice models which abstract away many of the details of protein folding.

Lattice protein models are simplified instances of the generic class of cooperative chain folding processes, which include the actual folding of biological macromolecules. Although these models cannot make actual predictions about real biological macromolecules, their fitness landscape and process dynamics share common traits with real-life processes and therefore may serve to characterize generic features of protein folding.

The hydrophobic-polar (HP) model [5] is a simplified model which has become a major tool for investigating general properties of protein folding. The HP model emphasizes hydrophobicity as the most important difference between amino acids considering two types of residues: H (hydrophobic or non-polar) and P (hydrophilic or polar). A sequence of such residues located in a lattice forming a self-avoiding chain represents a protein. Two residues are considered topological neighbors if they are adjacent (either horizontally or vertically) in the lattice and not consecutive in the sequence. The protein structure prediction problem in the HP model focuses on finding the protein configuration which minimizes the total energy. The energy function measures the interactions between topological neighbours as follows: each H-H topological contact contributes -1 to the energy while all the other interactions are not considered in the energy function. The protein conformation with minimum energy corresponds therefore to the protein configuration with the maximum number of H-H topological contacts. The HP model represents the simplest - yet non-trivial - abstraction for the protein structure prediction problem [2].

Existing computing approaches to protein folding in the HP model include evolutionary search [6-9], ant colony optimization [10], memetic algorithms [11], tabu search [12], Monte Carlo approximation algorithms [13] and constrained programming [14-16]. Although evolutionary algorithms have been extensively engaged as robust and efficient global optimization methods for this problem, their computing efficiency needs further improving. Evolutionary approaches to the protein structure prediction problem suffer from the limitations of the genetic operators for this particular problem search space. Crossover and mutation can easily produce invalid configurations due to potential collisions generated by changing various parts of a chromosome. This weak performance of standard genetic operators has a direct impact on the effectiveness of the evolutionary search process.

This paper presents an evolutionary model based on hill-climbing search operators designed to address the problem of protein structure prediction in the HP model. The proposed model evolves a population of protein configurations for a given HP sequence and relies on hill-climbing recombination and mutation to support the search process. Hill-climbing crossover is applied in a dynamic way and offspring are asynchronously inserted in the population during the same generation. The mutation operator engaged in the proposed model is problem-specific and is applied in a steepest-ascent hill-climbing manner. The basic mutation uses the pull-move transformation [12] by which a single residue is moved diagonally causing the transition of connecting residues. Hill-climbing mutation aims to improve a configuration by applying pull-move transformations in all possible positions. Both crossover and mutation ensure the generation of new valid configurations. The hill-climbing approach taken in this model enhances the exploitation capabilities of the search, vital for good results in protein folding problems. Furthermore, an explicit diversification stage engaged periodically during the population evolution replaces redundant individuals with new genetic material (generated based on the same process used in the population initialization phase). The aim of the diversification stage is to help the search escape local optima.

The experiments presented in the current paper focus on various bidimensional HP lattice protein sequences. We evaluate the proposed evolutionary model in two phases as follows: (i) the main model features - hill-climbing behaviour and diversification mechanism - are tested by comparing several variants of the proposed model in order to assess their impact to the efficiency of the algorithm, and (ii) the resulting best-performing version of the proposed evolutionary model is compared to relevant models from the literature for a set of difficult bidimensional HP instances. Numerical results indicate a competitive performance of the evolutionary algorithm based on hill-climbing operators.

The paper is organised as follows: related work on computational methods for addressing the protein structure prediction problem is presented, the proposed hill-climbing evolutionary model is described and assessed in a set of computational experiments and numerical comparisons are discussed.

## Computational Models for Protein Structure Prediction

The protein structure prediction problem for the HP model has been shown to be NP-hard [1,4] and many approximation methods and heuristics for addressing it have been proposed [1,2].

Local search methods rely on the idea of iteratively improving a protein conformation based on the exploration of its local neighborhood. However, traditional Monte Carlo methods for protein folding simulations easily get trapped in local optima due to the problem-specific characteristics of the search landscape. Chain growth methods have been proposed to cope with this problem. The pruned-enriched Rosenbluth method (PERM) [13] grows a sequence by sequentially adding one individual particle at a time. The growth is guided towards configurations with lower energies generating good results for the HP problem in 2D and 3D lattices. The main drawbacks refer to the need to incorporate heuristic knowledge and the usage of a significant number of weight thresholds [2].

Backofen and Will [14-16] present a constrained programming approach to protein structure prediction for the HP energy model on the cubic and face centered cubic lattice. The method combines branch-and-bound search with a constrain-and-generate principle to minimize the surface of the conformation (objective shown to be similar with maximizing the number of H-H contacts). Geometrical symmetries are excluded using binary branching trees. The authors show that transforming protein structure prediction to a constraint minimisation problem with finite domain variables guarantees finding the optimum conformation. However, the computation time required by constraint programming given various conformation sizes can become a limitation of the method.

Lesh et al [12] introduce a local search strategy called pull move for the bidimensional HP model. The pull move transformations are incorporated in a tabu search algorithm able to detect new lowest energy configurations for large HP sequences (having 85 and 100 amino acids). A pull move operation starts by moving a single residue diagonally to an available location. A valid configuration is maintained by pulling the chain along the same direction (not necessarily until the end of the chain is reached - a valid conformation can potentially be obtained sooner). The authors also prove that the class of pull moves introduced is reversible and complete [12].

Genetic algorithms (GAs) for protein structure prediction have been initially used by Unger and Moult [9] and proved to obtain better results than traditional Monte Carlo methods. Chromosomes are encoded using internal coordinates with absolute moves and a population of valid conformations is evolved by mutation and crossover. The performance of the 'simplest' genetic algorithm is investigated in [17] where the importance of high resolution building blocks (facilitated by multi-point crossovers) and of local dynamics operator is emphasized. A hybridization between GA and a backtracking algorithm is investigated in [7]. The use of a backtracking-based repairing procedure and of evolutionary search operators constraining the search to the space of valid conformations produces good results for the 3D HP problem. In [8], some specialized genetic operators (called symmetric and cornerchange operators) are introduced. The resulting GA is applied for HP sequences having a length up to 50 residues. In [6], the results of standard GA for protein structure prediction are improved by a GA using pull moves [12] as a local search genetic operation in addition to standard crossover and mutation.

Multimeme algorithms (MMAs) [11] combine GAs with a set of local search heuristics enforcing various neighborhoods for memetic algorithm search. In MMAs, each individual incorporates genetic and memetic material. Crossover, mutation, local search and replacement are performed each generation. MMAs further rely on a contact map memory of already visited solutions based on the topological features of the conformations. The MMA was successfully applied to both HP and functional model proteins.

The protein folding problem has also been tackled using nature-inspired metaheuristics which rely on the model of the search space (such as ant colony systems). Shmygelska et al [10] use Ant Colony Optimization (ACO) combined with a local search mechanism to construct protein conformations. Artificial ants iteratively construct solutions based on the quality of already determined solutions (through the indirect influence of pheromone updates in the search space).

## Proposed Evolutionary Model: Hill-Climbing Search Operators and Diversification Strategy

An evolutionary model relying on hill-climbing search operators to address the protein structure prediction problem is described. A chromosome represents a possible protein configuration for a given HP sequence. A population of configurations is evolved by hill-climbing crossover and mutation.

Offspring replace parents if they have a better fitness value. The search scheme is asynchronous in the sense that a new chromosome created as a result of crossover or mutation can potentially be exploited within the same generation by the search operators. Premature convergence is addressed by a *diversification scheme *in which similar individuals are identified and some of them are replaced by new genetic material. Besides the hill-climbing operators and the diversification scheme, the proposed evolutionary model does not require any other phase such as explicit selection or standard mutation. The distinct features of the introduced model can be summarized as follows:

1. The population size is fixed and offspring are asynchronously inserted in the population replacing the worst parent within the same generation.

2. Crossover is applied to randomly selected pairs of individuals in a hill-climbing mode. A number of *k *offspring are iteratively generated from the same parents. The best-fitted offspring (or its random hill-climbing mutation if better) replaces the worst parent within the same generation. If no better offspring is identified, both parents are replaced by new randomly selected chromosomes. The process continues until the maximum number of hill-climbing iterations is reached.

3. Mutation implements a steepest ascent hill-climbing procedure using the pull move operation [12]. This process is able to generate a variable number of new individuals which replace parents within the same generation (if they have a better fitness value).

4. Diversification ensures the existence of sufficiently heterogeneous genetic material by periodically checking the similarity between individuals having the same energy and replacing similar inidviduals with newly generated ones.

The general scheme of the proposed hill-climbing evolutionary model is given below.

   **Main Scheme of Evolutionary Algorithm based on Hill-Climbing Operators**

   Generate *P*(0) with *pop_size *individuals randomly

   **while **(maximum number of generations not reached) **do**

      Hill-climbing crossover for *k *offspring and *hc *iterations

      Hill-climbing mutation for *hc *iterations

      Diversification every *kd *generations

   **end while**

The evolutionary algorithm presented in this paper takes a standard approach to problem representation and fitness function in order to keep the emphasis of the obtained results on the hill-climbing genetic operators implemented.

A chromosome is encoded using an internal coordinates representation. For a protein HP sequence with *n *residues *S *= *s*_1 _*... s_n_*, the chromosome length is *n *- 1 and each position in the chromosome encodes the direction *L*(*Left*), *U*(*Up*), *R*(*Right*) or *D*(*Down*) towards the location of the current residue relative to the previous one.

The fitness function used corresponds to the energy value of the protein configuration.

### Hill-Climbing Mutation

The pull move operation proposed in [12] is used as a specialized mutation operator. A pull move transformation can be applied at a given position *i *from the considered HP sequence.

Let (*x_i_, y_i_*) be the coordinates in the square lattice of residue *i *at time *t*. Let *L *denote a free location diagonally adjacent to (*x_i_, y_i_*) and adjacent (either horizontally or vertically) to (*x*_*i*+1_, *y*_*i*+1_). Location *C *denotes the fourth corner of the square formed by the three locations: *L*, (*x_i_, y_i_*) and (*x*_*i*+1_, *y*_*i*+1_). A pull move is possible if location *C *is free or equals (*x*_*i*-1_, *y*_*i*-1_). In the latter case, the pull move transformation consists of moving the residue from location (*x_i_, y_i_*) to location *L*. In the case that *C *is a free location, the first step is to move residue from position *i *to location *L *and the residue from position (*i *- 1) to location *C*. The pull move transformation continues by moving all residues from (*i *- 2) down to 1 two locations up the chain until a valid configuration is reached.

**Figure 1:**
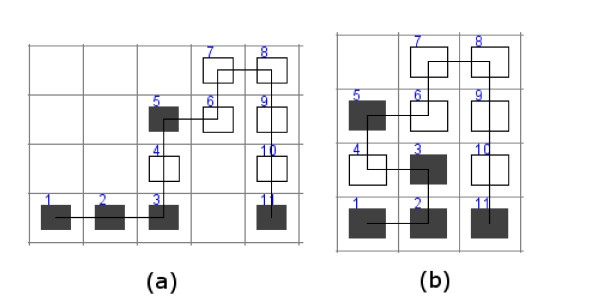
**Pull move transformation for HP sequence *HHHPHPPPPPH *represented by the chromosome *RRUURURDDD *(a)**. Part (b) represents the new chromosome *RULURURDDD *obtained after the pull move transformation at position 3. Figure 1 presents an example of a pull move transformation for HP sequence *HHHPHPPPPPH *having the chromosome value of *RRUURURDDD *(depicted in Figure 1a). Pull move is applied for residue *H *at position 3 and results in the chromosome value of *RULURURDDD *(Figure 1b).

Figure 1 presents an example of a pull move transformation for HP sequence *SE *= *HHHPHPPPPPH *having the chromosome value of *RRUURURDDD*. The pull move is applied for residue *H *at position *i *= 3 for which a free location *L *horizontally adjacent to residue *i *+ 1 (between residues 4 and 10 in Figure 1a is identified. Location *C *(the location between residues 3 and 11 in Figure 1a) is free in this example and therefore the pull move will cause moving the residue 3 to location *L *and residue 2 to location *C*. The remaining residue 1 (only one in this example) is moved up the chain two positions producing the new chromosome value of *RULURURDDD *(see Figure 1b).

In the proposed algorithm, pull moves are applied within a steepest ascent hill climbing procedure each generation. Hill-climbing mutation starts by randomly selecting one individual from the current population and setting it as the *current_hilltop*. Pull moves are applied at each position *i, i *= 1, ..., *n *(where *n *is the length of the HP sequence) resulting in the generation of *n *new chromosomes. If any of them has a better fitness value than the *current_hilltop *it replaces the latter one. If no improvement is achieved and the maximum number of hill-climbing iterations has not been reached, the *current_hilltop *is reinitialized with a new individual randomly selected from the population.

The procedure for hill-climbing mutation is given below.

Hill-Climbing Mutation Procedure

Set *current hilltop *to a randomly selected individual *rand_c*

Set *best_c *to *current_hilltop*

**while **(maximum number of *hc *iterations not reached) **do**

   **for ***i *= 1 to *n ***do**

      Generate new chromosome *c_i _*by applying a

      pull move transformation at position *i *in *current_hilltop*

      **if **(*c_i _*has better fitness than *best_c*) **then**

         Set *best_c *to *c_i_*

      **end if**

   **end for**

   **if **(better chromosome *best_c *found) **then**

      Set *current_hilltop *to *best_c*

   **else**

      Replace *rand_c *with *best_c *in the current population

      Set *rand_c *to a new randomly selected individual

      Set *current_hilltop *and *best_c *to *rand_c*

   **end if**

end while

It should be emphasized that the number of individuals that will undergo hill-climbing mutation within one generation is dynamic. The hill-climbing mutation procedure operates on the same individual by pull move mutation until no further improvement is achieved. The mutated chromosome obtained in this process replaces the original parent in the population during the current generation. The hill-climbing mutation procedure continues with a new individual randomly selected. The process of improving a chromosome by pull moves can last a variable number of hill-climbing iterations for each individual.

### Hill-Climbing Crossover

For the recombination of genetic material, a one-point crossover operator is specified. Given two parent chromosomes *p*_1 _and *p*_2 _and a randomly generated cut point *χ*, two offspring are created as follows:

1. The genes before the cut point *χ *are copied from one parent: , for *i *= 0, *χ *- 1;

2. For the second part of the offspring, , for *i *= *χ, n *- 1 unless this move forces residue *i *to overlap with one of the *i *- 1 previous ones. If a collision occurs then a random direction leading to a valid position is selected.

**Figure 2:**
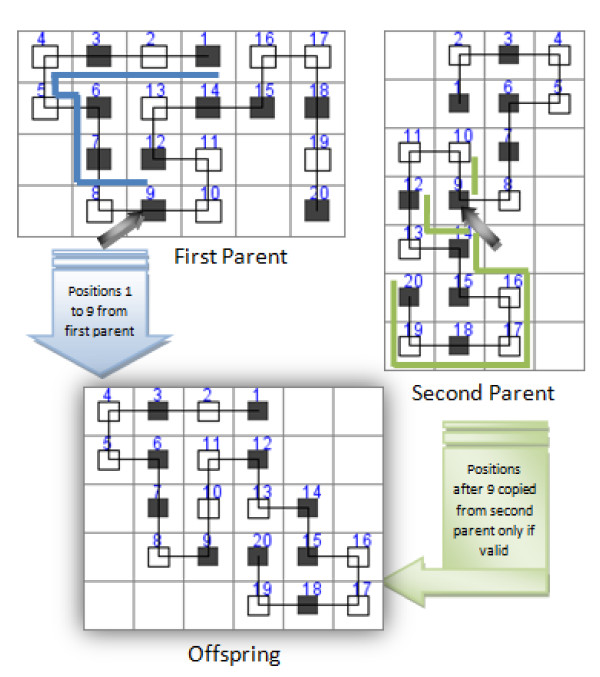
**Dynamic crossover example**. Figure 2 presents an example of crossover applied to a pair of chromosome parents of size 20. The crossover point is after position 9 which means that the offspring inherits values at positions 1 to 9 from the first parent while the remaining positions are completed in a dynamic way (in order to avoid collisions) using values from the second parent.

Figure 2 presents an example of how the proposed crossover operator works.

Crossover is applied following a hill-climbing strategy. In every generation, a variable number of chromosome pairs are selected for crossover and better generated offspring replace individuals in the current population.

The hill-cllimbing crossover procedure is detailed below. This procedure is inspired by the crossover-hill-climbing scheme proposed in [18].

 **Hill-Climbing Crossover Procedure**

 Set *p*_1 _and *p*_2 _to randomly selected individuals from current population

 Set *best_o *to *best*(*p*_1_*, p*_2_)

 **do**

   **for ***i *= 1 to *k ***do**

      Generate a random cut point *χ *(from 1 to chromosome length *n *- 1)

      Set *o *to the best of two offspring obtained from *crossover*(*p*_1_*, p*_2_*, χ*)

      **if **(*o *has better fitness than *best_o*) **then**

         Set *best_o *to *o*

      **end if**

   **end for**

   **if **(new *best_o *found) **then**

      Set *rhcm_best *to *random_hill_climbing_mutation*(*best_o*)

      Replace *worst*(*p*_1_*, p*_2_) with *best*(*best_o, rhcm_best*)

   **else**

      Set *p*_1 _and *p*_2 _to new individuals randomly selected

      from current population

   **end if**

 **while **(maximum number of *hc *iterations not reached)

For each pair of chromosomes selected for recombination, a number of *k *offspring is generated via crossover. The best offspring resulted from this process is mutated using a pull move transformation within a random hill climbing (RHC) procedure. This RHC mutation is similar to the hill-climbing mutation presented in the previous section but it has the following distinctive features: (i) only one chromosome is being mutated and when no further improvement is obtained by pull moves the procedure stops; and (ii) at each hill-climbing iteration, only one pull move transformation is applied for a position randomly selected. The parent having the highest energy is replaced with the best of the two chromosomes generated (best offspring from crossover and its RHC mutated version). This new individual is engaged as a parent in the next hill-climbing iteration. When no better offspring is generated, a new pair of parent chromosomes is randomly selected from the current population and undergoes the same steps until the maximum number of hill-climbing iterations is reached. Similar to the mutation procedure, the number of individuals selected for recombination varies from one generation to another depending on the improvements that can be generated by the same pair of chromosomes.

### Diversification

In order to ensure the maintainance of sufficiently diverse genetic material, it is proposed to explicitly reinforce diversity every *kd *generations, where *kd *is a parameter of the algorithm. The diversification stage works as follows:

1. The individuals from the current population are grouped based on their fitness (one group for each fitness value).

2. For each group identified, subgroups of similar individuals are constructed based on the Hamming distance. Individuals are considered similar if the Hamming distance (i.e. the number of different position values in the chromosomes) is less than (*n *- 1)*/*4, where (*n *- 1) is the length of the chromosome.

3. For each subgroup of similar individuals, one of them is kept in the current population and the rest of individuals are replaced by new randomly generated chromosomes (improved by a hill-climbing mutation).

Diversification has the potential to avoid the search process to get trapped in local optima by explicitly introducing new genetic material in the population. The exploration of new search space regions is therefore facilitated in addition to the efficient exploitation performed by the hill-climbing procedures.

## Evaluation of the Main Proposed Model Components

The main features of the proposed evolutionary model refer to the hill-climbing mechanism applied to the search operators and the diversification strategy engaged periodically during the evolution of the population. These features are assessed in a set of numerical experiments in order to determine their relative importance in the search process and impact on the efficiency of the algorithm for the protein structure prediction problem in the HP protein model.

### Compared Model Variants and Parameters

The following four algorithms are engaged in a set of numerical experiments (going top-down from the main proposed model presented in the previous section) for a comparative analysis:

• *EAHCD (Evolutionary Algorithm with Hill-Climbing search and Diversification) *represents the evolutionary model which includes hill-climbing genetic operators and the diversification mechanism (the exact same model presented in the previous section).

• *EAHC (Evolutionary Algorithm with Hill-Climbing search) *represents the evolutionary model based on hill-climbing search but with no diversification mechanism engaged.

• *EAD (Evolutionary Algorithm with Diversification) *represents the evolutionary model which includes the diversification strategy but does not apply the hill-climbing mechanism to the genetic operators.

• *EA (Evolutionary Algorithm) *is the most simple evolutionary model where neither hill-climbing search operators or diversification mechanism are engaged.

The latter two algorithms presented in the above list use the same basic crossover and mutation operators (described for the model in the previous section) but they are not being reinforced within a hill-climbing search procedure. Instead, crossover is applied with probability of 0.8 for each individual of the population and a mate is selected using binary tournament. Mutation probability is 0.2 for each individual in the population. For all algorithms, the population size is 100 and the number of generations is 300. For EAHCD and EAHC, the number of hill-climbing iterations for both crossover and mutation is 100. For EAHCD and EAD, diversification is engaged every generation.

### Benchmark Protein Sequences

Table 1 presents 2D HP instances considered for the computational experiments. For each HP sequence, the known optimum value is given in the column labelled E*.

**Table 1:** Standard 2D HP instances used as benchmark

**Inst**.	Length	Sequence	E*
S1	20	1H 1P 1H 2P 2H 1P 1H 2P 1H 1P 2H 2P 1H 1P 1H	-9
S2	24	2H 2P 1H 2P 1H 2P 1H 2P 1H 2P 1H 2P 1H 2P 2H	-9
S3	25	2P 1H 2P 2H 4P 2H 4P 2H 4P 2H	-8
S4	36	3P 2H 2P 2H 5P 7H 2P 2H 4P 2H 2P 1H 2P	-14
S5	48	2P 1H 2P 2H 2P 2H 5P 10H 6P 2H 2P 2H 2P 1H 2P 5H	-23
S6	50	2H 1P 1H 1P 1H 1P 1H 1P 4H 1P 1H 3P 1H 3P 1H 4P 1H 3P 1H 3P 1H 1P 4H 1P 1H 1P 1H 1P 1H 1P 1H 1H	-21
S7	60	2P 3H 1P 8H 3P 10H 1P 1H 3P 12H 4P 6H 1P 2H 1P 1H 1P	-36
S8	64	12H 1P 1H 1P 1H 2P 2H 2P 2H 2P 1H 2P 2H 2P 2H 2P 1H 2P 2H 2P 2H 2P 1H 1P 1H 1P 12H	-42

The evaluation phase consists of running each of the four compared variant models 25 times for each HP sequence considered. For each run, the identification of a better solution is timestamped offering the grounds for comparison.

### Evaluation Tools

In the analysis presented in this section, *n_A_*_@*T *_represents the number of instances having already produced a best-to-date offspring at time T for an algorithm A. Plots of time T report the following:

• *min*(*E*)*_A_*_@*T *_: minimal energy ever obtained at time T for algorithm A - bar bottom,

• *avg*(*E*)*_A_*_@*T *_- *var*(*E*)*_A_*_@*T *_: average best-to-date energy minus variance - rectangle bottom,

• *avg*(*E*)*_A_*_@_*_T _*+ *var*(*E*)*_A_*_@*T *_: rectangle top, and

• *max*(*E*)*_A_*_@_*_T _*: largest best-to-date energy - bar top.

At time T, the Student statistical parameter *t *[19] is used to check whether the distribution of best-to-date energies of two algorithms A1 *n_A_*_1@*T*_, *avg*(*E*)*_A_*_1@*T*_, *var*(*E*)*_A_*_1@*T *_and A2 *n_A_*_2@*T*_, *avg*(*E*)*_A_*_2@*T*_, *var*(*E*)*_A_*_2@*T *_are significantly different. The curves labeled *A*1 - *A*2 display signed *t *values: if [*avg*(*E*)*_A_*_1@*T *_± *var*(*E*)*_A_*_1@*T *_] ≫ [*avg*(*E*)*_A_*_2@_*_T _*± *var*(*E*)*_A_*_2@*T *_] then *t*(*T*) is large and positive - i.e. algorithm A1 showed significantly poorer results at time T compared to algorithm A2. By contrast, if *t*(*T*) is strongly negative, then algorithm A1 is better - eventually, at *t*(*T*) ≈ 0 both methods are performing equally well. It should be noted that that the *Y *axis is labeled in terms of *t *values and the actual energy range values covered by the error bars are not shown.

## Results and Discussion

**Figure 3:**
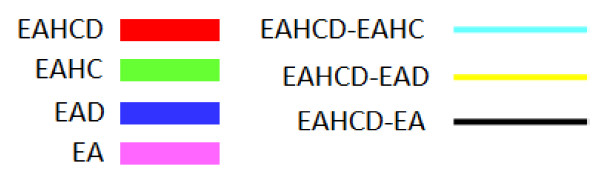
**Legend for the results presented in Figure 4**. Figure 3 presents the legend used for the comparative results given in Figure 4. It depicts the representation of compared *EAHCD, EAHC, EAD, EA *algorithms and the lines used for the statistical comparisons between pairs of algorithms.

**Figure 4:**
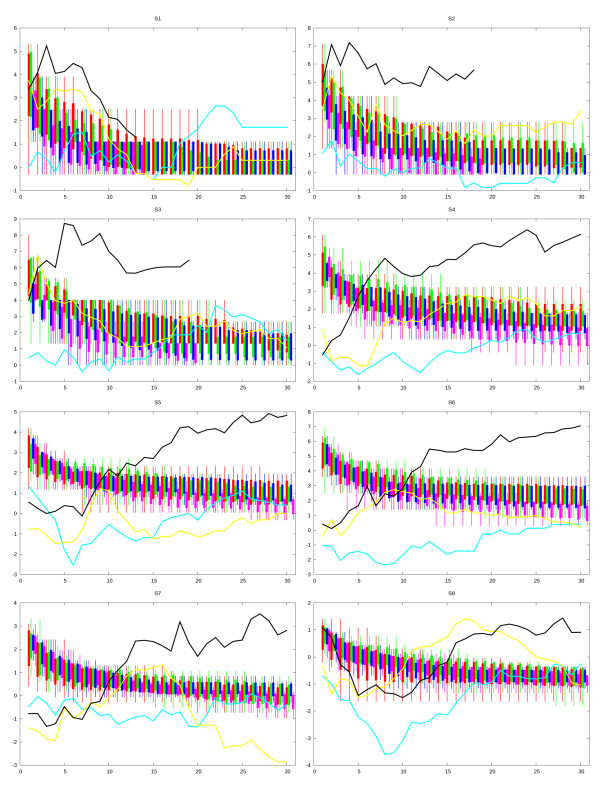
**Comparative results obtained by EAHCD, EAHC, EAD and EA for each of the eight HP sequences**. Figure 4 presents the results obtained for various HP sequences and shows the *EAHCD***-***EAHC*, *EAHCD-EAD *and *EAHCD-EA *statistical comparison curves.

The legend used for presenting the results is given in Figure 3 showing the representation of compared *EAHCD, EAHC, EAD, EA *algorithms and the lines used for the statistical comparisons between pairs of algorithms. Figure 4 presents the results obtained for HP sequences S1 to S8 from Table 1 according to the legend given in Figure 3. For each sequence, the *t *values comparing the most comprehensive algorithm *EAHCD *with the other three algorithms considered are shown - therefore the *EAHCD-EAHC, EAHCD-EAD *and *EAHCD-EA *statistical comparison curves are depicted.

As a general trend, a difference in behaviour of the algorithms for different sequences is clearly observed. For the smaller sequences considered the most simple variant algorithm - *EA *- has the best performance while *EAHCD *and *EAD *are slower. The benefits of using diversification and hill-climbing search become obvious with the higher size sequences considered - particularly S6, S7 and S8 - where the search space is more complex and a simple evolutionary approach easily gets trapped in local optima. Nevertheless, even in these latter cases, hill climbing is not necessarily needed as the performance of EA - except for sequence S8 - seems to remain competitive. In terms of best energy values achieved at the end of the run, *EAHCD *is the only algorithm able to identify best solutions for sequences S5, S7 and S8. Longer sequences still will have to be analyzed, in order to ascertain that the emerging trend may be generalized and shown to actually lead to an inversion of relative performance.

Towards the end of the monitored run time range, *EA *and *EAD *reproducibly reach some suboptimal energy level, and seem unable to "escape" this local optimum. The hill climbing strategies sporadically manage to discover deeper energy minima at comparable times. Interestingly, the standard deviation values of the best-so-far energies at time *t *seem to be less dependent on whether the diversification mechanism is turned on or off, but more on the use of hill climbing. For example, in 113 instances - over all sequences, at all times, monitored in the Figure 4, the standard deviation of *EAHCD *exceeds the corresponding *EAHC *value, whereas in 111 instances, the spread of the best energies obtained at time *t *with *EAD *exceeded the one produced by *EA*. Diversification within the population does therefore seem to trigger a diversification of the exploratory paths taken by the algorithms in problem phase space, and therefore add more randomness to the expectation value of the best-to-date energy at any point in time. However, there are 141 instances in which *EAHCD *produced broader scattering of these values compared to *EAD*, and 146 witnessing *EAHC *scatters being broader than equivalent *EA *output. Accordingly, hill climbing is a major source of diversification. Apparently, the aggresive attempts to find the best possible offspring/mutant lead, more often than in the classical evolutionary algorithms, to successful moves in phase space.

The present work followed the established "tradition" to monitor performance in terms of best-to-date energies only [9,6,11], but the current results clearly suggest that such an accounting does not properly capture the intrinsic sampling quality of the algorithms. Not only the best optimum, but all relevant suboptimal configurations sampled at time t should have been monitored - a tentative suggestion in this sense would be to use, instead of the best-to-date energy, the free energy level of the population *F *= -*kT *ln *Z*, where *k *is the Boltzmann constant, *T *the temperature and *Z *the partition function of the population, i.e. the sum of the Boltzmann terms *e*^-*E/kT *^of all the conformers in the population. This is well in line with the multimodal aspect of protein structure prediction, where failure to sample suboptimal, but populated conformers may prevent understanding of biological mechanisms involving protein flexibility.

### The Influence of the Hill-Cimbing Iteration Number

**Figure 5:**
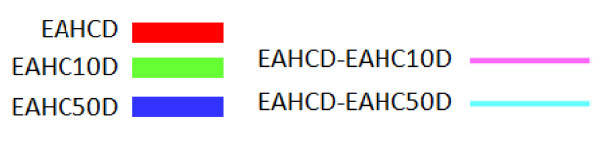
**Legend for the results presented in Figure 6**. Figure 5 gives the legend used in presenting the hill-climbing comparative results from Figure 6.

**Figure 6:**
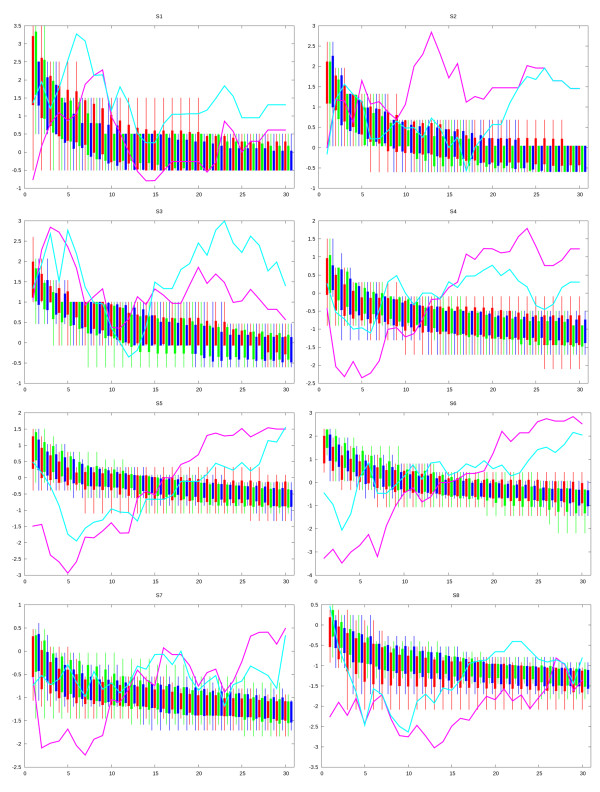
**Comparative results obtained by EAHCD with 10, 50 and 100 hill-climbing iterations**. Figure 6 compares the performance of the proposed algorithm with different values for the number of hill-climbing iterations: 10 - algorithm *EAHC10D*, 50 - algorithm *EAHC50D *and 100 - algorithm *EAHCD*.

All the tests presented above use 100 hill-climbing iterations for applying recombination and mutation in the *EAHCD *model. In this section, we intend to analyse the importance of this parameter by comparing the performance of the *EAHCD *algorithm with different values for the number of hill-climbing iterations: 10 (algorithm called *EAHC10D*), 50 (*EAHC50D*) and 100 (corresponding algorithm is the already analysed *EAHCD*). The legend used in presenting the results is given in Figure 5 and the results obtained are given in Figure 6.

These experiments confirm the need for more hill-climbing iterations only for the higher-size HP sequences (S5, S6, S7 and S8) where 100 iterations lead to a better search space exploitation compared to 10 or 50. For less complex search spaces (such as S1, S2, S3) the algorithms with less (or none) hill-climbing iterations are still able to produce good solutions both in terms of energy values and running times.

## Numerical Experiments and Comparisons with Related Methods

Comparative numerical experiments focus on the 2D HP protein sequences (commonly used as benchmarks) with lengths from 20 to 64 given in Table 1.

Due to an overall best performance based on energy values detected, the *EAHCD *variant model has been engaged in these computational experiments. The parameter setting for the proposed algorithm is the following:

1. The population size is 100 and the number of generations is 300;

2. The number of hill-climbing iterations for both crossover and mutation is set to 100;

3. For hill-climbing crossover, a number of 50 offspring are generated for a pair of chromosomes each hill-climbing iteration;

4. Diversification is engaged every 30 generations (generally calculated as 10% of the number of generations).

The initial population contains randomly generated chromosomes representing valid configurations (each chromosome is iteratively generated in a random manner until a conformation free of collisions in the HP square lattice model is found). For each HP sequence considered, the proposed algorithm was run 10 times and the results from one of the most efficient runs are reported.

The performance of the proposed model is compared to the best results obtained by other evolutionary models and memetic algorithms for protein structure prediction. Table 2 presents the results as follows: the known optimum for each HP instance, the energy found by the proposed method (in the third column), the results of standard GA [9], Pull-Move GA (PMGA) [6] and multimeme algorithms (MMA) [11]. The results of GA [9] are based on a population of 200 structures evolved over 300 generations. The Pull-Move GA (PMGA) proposed in [6] is able to improve the results of standard GAs by using pull move transformations in addition to the standard genetic operators but not in a hill-climbing mode. MMAs represent an interesting approach to be compared with the one proposed in this paper as both models use local search but according to different strategies. For GA and MMA the best solution from 5 runs is selected while the PMGA reports the best solution from 10 runs.

**Table 2:** Comparative results for the considered HP sequences

**Inst**.	Length	E*	**Proposed Method (EAHCD)**	**Genetic Algorithms**	Pull Move-GAs	**MultimemeAlgorithms**
S1	20	-9	-9	-9	-9	-9
S2	24	-9	-9	-9	-9	
S3	25	-8	-8	-8	-8	-8
S4	36	-14	-14	-14	-14	-14
S5	48	-23	-23	-22	-22	-22
S6	50	-21	-21	-21	-21	-21
S7	60	-36	-35	-34	-34	
S8	64	-42	-39	-37	-38	-39

The *EAHCD *model is able to identify the protein configurations having the best known optimum energy for sequences *S*1 to *S*6. For the first three HP instances considered, optimum energy conformations are found very early in the evolution process (usually during the first 20 generations) and therefore, a less computationally expensive implementation of the algorithm (with fewer generations and probably less hill-climbing iterations) would have been able to generate the optimum. Indeed, the more simple model variant *EA *(analysed in the previous section) proved to be highly succesful in dealing with small-size HP protein sequences. The proposed algorithm fails to find the optimum for the larger instances *S*7 and *S*8. Most of the runs of the algorithm for sequence *S*7 detect the suboptimal solution having the energy -35. We expect to improve the performance of the proposed model for large instances by extending the diversification stage to consider other metrics for calculating the similarity between two chromosomes. For example, a *fingerprint *of the protein configuration (which includes topological information) can potentially provide a more accurate comparison between same-energy individuals so that the diversification stage would result in the replacement of meaningfully similar chromosomes.

A direct comparison between energy values obtained by different evolutionary models emphasizes a good and competitive performance of the proposed method. The results are better than those of GAs [9] and PMGAs [6]. The effect of hill-climbing search based on pull moves is clearly benefic as opposed to applying pull moves in addition to mutation as in PMGA (the proposed model detects better energies for instances *S*5, *S*7 and *S*8 compared to PMGA). The results are competitive with those of MMA (the proposed model obtains a better solution for instance *S*5 when compared to MMA). This is a promising result for the proposed method emphasizing the power of hill-climbing search procedures based on specialized genetic operators. In MMAs, optimization is based on the memes available individually (pivot moves, substructure stretching, random macro-mutation of a substructure, reflection of a sub-structure, non local *k*-opt and local *k*-opt) [11]. The proposed model uses a scheme by which a dynamic number of individuals are affected each generation by hill-climbing search operators and is able to detect similar or better results compared to MMAs where optimization (based on the six memes mentioned above) is applied for every individual in the population in addition to standard evolutionary search.

## Conclusions and Future Work

An evolutionary model based on a hill-climbing search scheme and diversification for the protein structure prediction in the HP model is presented. The main feature of the proposed model refers to the application of crossover and pull move transformations (as mutation) within hill-climbing search procedures. Better offspring are inserted in the population within the same generation making the selection process intrinsic to hill-climbing crossover and mutation. An explicit diversification process is engaged periodically to replace similar chromosomes with new genetic material.

The extensive analysis of the various evolutionary model variants suggests the need for more robust evaluation mechanisms of computational models for protein structure prediction. In this sense, it would be well-worthed to monitor all relevant suboptimal configurations sampled at time t and not only the highest-energy conformations.

The results obtained by the proposed model for several bidimensional HP instances are promising and competitive with the best results of other evolutionary models. Future work focuses on the extension of the proposed model for the 3D HP protein model. More complex approaches to the calculation of the fitness energy will also be investigated. Furthermore, the proposed evolutionary model can highly benefit from the improvement of diversification using other mechanisms for checking the similarities between individuals (such as the fingerprint of protein conformations).
